# When a COVID-19 vaccine is ready, will we all be ready for it?

**DOI:** 10.1007/s00038-020-01404-4

**Published:** 2020-06-11

**Authors:** Marta Fadda, Emiliano Albanese, L. Suzanne Suggs

**Affiliations:** Institute of Public Health, Università Della Svizzera Italiana, via Buffi 13, 6900 Lugano, Switzerland

The current response to the COVID-19 pandemic involves aggressive implementation of containment, suppression, and mitigation strategies. Such an approach encompasses the enforcement of a variety and combination of public health measures including hand hygiene and respiratory etiquette, disinfection, case identification, isolation of sick people, tracing and quarantine of contacts, and unprecedented mass community restrictions. Besides considerable investments in interventions to contain transmission, and in diagnostics and therapeutics research, whose way forward is framed within the WHO coordinated global research roadmap (WHO 2020), a vaccine represents the most promising strategy for combatting the COVID-19 pandemic through primary prevention. In addition to bio-tech companies and governments pushing, and in some cases suggesting, that a vaccine may be available as early as Fall 2020, it is widely believed that an effective and available vaccine will be ready for licensure no sooner than 12–18 months from now (Kormann 2020). Five phase I clinical trials are ongoing in the USA and/or China and other trials are expected to be initiated soon in Germany and the UK (Le et al. 2020).

The sole availability of a vaccine does not equal uptake. For example, in 2009, despite a vaccine against influenza A H1N1 being offered before or at the onset of the second epidemic wave, vaccination rates were lower than expected, with population coverage ranging from 0.4 to 59% across 22 countries (Mereckiene et al. 2012). The low uptake of an available vaccination for a high risk infection has been called a “pandemic public health paradox” (Reintjes et al. 2016), to which vaccine hesitancy (i.e. “the reluctance or refusal to vaccinate despite the availability of vaccines”) significantly contributes. While hesitancy level and reasons vary by vaccine, geographic location, health system, accessibility and availability and can be driven by emotional, cultural, social, and political factors as much as cognitive ones (MacDonald et al. 2015), one characteristic is universal; vaccines only protect if enough people get them.

As the world is focused on combatting COVID-19 by imposing protective and preventive measures, the expectation is that an effective vaccine will come to market and allow us to contain the spread of the virus. However, vaccine availability is necessary but not sufficient to attain a significant reduction of susceptible individuals through active acquired immunity. Public opinion and trust in the vaccine is of paramount importance for adequate coverage. The current pandemic entails three unique challenges for public confidence in and uptake of a future, licensed vaccine. First, evidence shows that the newer the vaccine is, the level of hesitancy is higher (Dubé et al. 2013). Knowledge and understanding of the disease, and the reputation of vaccine developers contribute to trust and mis-trust. However, public information on the specific SARS-CoV-2 antigen(s) used in vaccine development is limited, and the majority of the confirmed active vaccine candidates are being developed by small and/or less well-known manufactures (Le et al. 2020). Second, one reason people trust vaccines is the slow and methodical process it takes to develop them, which may take up to several years before final approval. The expedited approval of a new COVID-19 vaccine may counteractively contribute to hesitancy grounded on the public impression that the vaccine was rushed to the market and not sufficiently tested for both safety and efficacy. A third challenge relates to falsities and misinformation propelled by anti-vaccination campaigners. In fact, these campaigners are already active online and in their communities spreading unsupported opinions and distorted information about the virus and any vaccine that may eventually be offered. Studies are demonstrating that COVID-19 vaccine hesitancy level varies from low to high, with some 29% of New York residents claiming they will refuse a vaccine, compared to 20% of those in Canada (Latimer 2020) and 6% of those in the UK (Henley et al. 2020).

While COVID-19 vaccines are under development, the nature and extent of vaccine hesitancy must be assessed and ultimately addressed. For ongoing and future vaccine hesitancy studies, there is an opportunity to included COVID-19 specific items, and we strongly encourage this route. Accordingly, countries can use this evidence to design and implement strategic, targeted and tailored communication and policies aimed at promoting confidence in and demand for a COVID-19 vaccine. As described by the WHO Sage working group on vaccine hesitancy, following a social marketing approach is recommended to tackle vaccine hesitancy (Thomson et al. 2018). Such an approach should be grounded on existing evidence, and include a participatory approach with ongoing community engagement, to understand needs as they develop and change. Guided by existing frameworks for communication and message design, the information should inform communication that resignates with individuals, is relevant, timely, understandable and provided through channels and messengers that they trust. As with other vaccines (Opel et al. 2015), communication training of healthcare providers and other key stakeholders in the immunisation landscape is essential.
